# Middle-Aged and Older African Americans' Information Use During the COVID-19 Pandemic: An Interview Study

**DOI:** 10.3389/fpubh.2021.709416

**Published:** 2021-10-07

**Authors:** Lu Tang, Felicia N. York, Wenxue Zou

**Affiliations:** Department of Communication, Texas A&M University, College Station, TX, United States

**Keywords:** COVID-19, African Americans, information use, interview, health disparities

## Abstract

African Americans in the United States have been disproportionately affected by the COVID-19 pandemic in infection and mortality rates. This study examined how middle-aged and older African American individuals accessed and evaluated the information about COVID-19. Semi-structured interviews with 20 individuals (age: 41–72) were conducted during the first stay-at-home advisory period in late March and early April 2020. The phronetic iterative approach was used for data analysis. We found that these individuals primarily relied on information scanning based on their routine media consumption to acquire information about COVID-19 and seldom actively searched for information outside of their regular media use. Individuals used several strategies to assess the quality of the information they received, including checking source credibility, comparing multiple sources, fact-checking, and praying. These findings could inform media and governmental agencies' future health communication efforts to disseminate information about the COVID-19 pandemic and future infectious disease outbreaks among the African American communities.

## Introduction

The COVID-19 pandemic has disproportionately affected racial and ethnic minorities in the United States, especially African Americans^1^. According to the Centers for Disease Control and Prevention (the CDC), up to June of 2020, 21.8% of confirmed COVID-19 cases in the U.S. were African Americans, although this racial group only accounts for 13% of the U.S. population (1). The statistics provided by The Johns Hopkins University and American Community Survey suggested that the infection rate and the death rate for the predominantly black counties were 137.5/100,000 and 6.3/100,000, respectively (2). The infection rate in predominantly black counties was three times the rate in predominantly white counties. More importantly, the death rate in predominantly black counties was six-fold higher than that for predominantly white counties (3).

Several factors have contributed to the high morbidity and mortality rate among African American communities (3). African Americans are more likely to have pre-existing conditions, such as hypertension, diabetes, obesity, and cardiovascular disease (3). In addition to comorbidity, African Americans have less access to quality healthcare (4). They are also more likely to live in less affluent neighborhoods with high housing density (3). Furthermore, African Americans are more likely to have jobs that do not allow social distancing or telecommuting.

Another important contributing factor to racial health disparity is health literacy. Individuals of racial and ethnic minority and lower socioeconomic groups are often burdened with low health literacy (5). Past research has shown that African Americans have more information insufficiency (i.e., the discrepancy between the information people desire and the information they have) (6) and are less likely to seek information about many health topics (7). How African Americans get information about COVID-19 might influence their risk perceptions and prevention behaviors. This study explores how middle-aged and older African Americans acquire and evaluate the information about COVID-19 during the outbreak's initial stage.

## Literature Review

Health literacy is “the degree to which individuals can obtain, process, and understand basic health information and services need to make appropriate health decisions” (8). Health literacy is more than general literacy in that it includes the ability of information seeking, critical information analysis, and decision making (9). According to the social ecological model of health literacy, health literacy is influenced by external factors in the physical and social environment such as individual, interpersonal, organizational, community, and policy factors (10). This model facilitates the understanding of disparity in health literacy.

During public health crises, individuals need the information to understand their risks and plan their behaviors. Several theoretical models explain the factors affecting how people acquire and process risk information. According to the Planned Risk Information Seeking Model (PRISM), individuals' information-seeking intentions and behaviors are influenced by cognitive factors (e.g., perceived risks, existing knowledge), affective factors (e.g., emotional response to threats), and subjective norms about information seeking (11). The Risk and Information Seeking and Processing Model (RISP) suggests that increased risk perceptions will trigger negative emotional responses, leading to an assessment of information needs and subsequent information seeking and processing (12). A meta-analysis of empirical studies using the RISP and PRISP models showed that current knowledge and subjective norms about information seeking are the two most important predictors of information seeking and systematic processing (13).

### Health Information Use During Public Health Crises

Individuals acquire health information through information-seeking and information scanning. Information seeking happens when individuals intentionally look for a certain kind of information (e.g., reading a newspaper article or searching for a topic on the Internet). Information scanning occurs when individuals receive information during their routine media consumptions (e.g., watching TV, mobile phone push notifications) (14). Information seeking requires more effort than information scanning.

In health communication, information seeking includes proactive behaviors such as looking for information about an illness using search engines, asking for information during conversations with physicians, and reading articles about different health topics in newspapers or the Internet. Information scanning involves being exposed to health and illness information during one's routine media consumption, such as watching TV, listening to the radio, flipping through a magazine in a doctor's office, and looking at information pushed by social media apps. Health information scanning is likely to expose people to multiple sources of information, but health information seeking is more likely to change people's behaviors (15).

Health communication scholars have examined how people acquire information during public health crises such as outbreaks of infectious diseases. Van Velsen et al. (16) surveyed college students in the Netherlands about their information use during an *E. coli* outbreak in Europe. They found that college students were most likely to use and trust news websites and websites of newspapers, and they tended to distrust social media and used them less frequently for information related to the outbreak. Randle et al. (17) studied how residents of Ontario, Canada looked for information during the Zika virus outbreak using Google Trends and telemedicine service data and found that both Google search and telemedicine consulting peaked when the WHO declared Zika a Public Health Emergency of International Concern (PHEIC) in February and then dropped precipitously. The public's information-seeking remained low until a second smaller Peak occurred in August when CDC issued a travel advisory for pregnant women to Miami. Recently, in the context of the COVID-19 outbreak, Tang and Zou (18) examined the media consumption of residents of Hubei Province, which was the epicenter of the COVID-19 outbreak in China, and found that they predominantly relied on social media for health information and governmental sources were most trusted.

### Health Information Processing

Researchers have examined how people process health information. One of the prominent theoretical models used was the heuristic-systematic model. This model identifies two information processing modes: systematic and heuristic. Individuals engage in systematic processing when they try to understand and evaluate an argument by investigating the facts used and analyzing the internal logic of the argument. In contrast, if they process an argument through peripheral cues such as source credibility and membership, they are engaging in heuristic processing (19). Systematic processing is cognitively more demanding than heuristic processing. People's use of these different processing modes is motivated by the need for accuracy, the desire to form judgments consistent with one's prior beliefs and values, and the need to fulfill one's social goals (20).

The HSM has been used to study how people process health and risk communication. Griffin et al. (21) studied how people sought and processed health and environmental risk information and found that individuals were more likely to engage in systematic processing when they had strong attitudes toward the issue and high evaluation strength (the degree to which they agreed with an evaluation of the risk). Kahlor et al. (22) found that the perceived amount of information needed was positively related to systematic processing. In other words, when individuals believe there is a gap between what they know and what they need to know, they are more likely to think about the messages deeply. The HSM can also guide an exploration of how African American individuals seek and process the information related to the COVID-19 pandemic.

### African Americans' Health Information Use and Communication Disparity

In the United States, racial and ethnic minorities have experienced different health disparities. Health disparities have been attributed to differences in genetic vulnerability to illnesses, access to health resources, and living conditions (23). The unequal access to health information and difference in information-processing behaviors is another factor contributing to health disparity (24). For instance, Laz and Berenson (7) surveyed young women (16–24 years of age) and found that Black and Hispanic women were less likely to use the Internet for health information seeking than white women in general (7). However, Hovick et al. (6) conducted a phone survey of low-income African American and white women in the southern United States and found that while African American women had greater information insufficiency, they were more likely to engage with systematic processing than white women.

Besides information seeking and processing, differences in trust in information sources and channels across racial and ethnic groups have been noted in previous studies. For instance, Nguyen and Belgrave (25) found that minority groups, including African Americans, Asian Americans, and Hispanics, usually prioritized informal interpersonal communication within the community and religion-based organizations when choosing health information channels. A study based on the Health Information National Trends Survey (HINTS) data revealed that Black or African Americans were more likely to trust religious organizations and religious leaders than whites, and Hispanics have lower trust in health providers than whites (26). Oh et al.'s study (27) examined the same HINTS dataset and concluded that minority groups had more faith in radio and television than whites; meanwhile, Blacks or African Americans trust governmental institutions more than whites. At this moment, there is little research on how African Americans access COVID-19 related information and how they evaluate such information. It is within this context that we propose the following two research questions (RQs):

*RQ1: How do African American individuals seek and scan information during the COVID-19 outbreak?*
*RQ2: How do African American individuals evaluate the information about COVID-19 they receive?*

## Methods

We designed a semi-structured interview study to explore how African American individuals acquire and evaluate information related to the COVID-19 pandemic and gain an in-depth understanding of the reasons they gave for their choices.

### Participants Recruitment

Convenience and snowball sampling was used. After the study was approved by the Institutional Review Board at the authors' institution, the second author reached out to her family and friends via social media, phone calls, and text messages to recruit potential participants. Those family and friends also referred other friends for the interviewer to contact and recruit. The inclusion criteria used initially were (1) must be African American, and (2) must live in the US at the time of data collection. We tried to achieve a balance of men and women in recruitment as well. Twenty-two participants completed the interview. After the completion of interviews, we found out that one participant was Caucasian and excluded the said participant from the study. In addition, we realized that among the remaining 21 African American participants, 20 of them were over 40 years old, and only one participant was in his early 20's. In this case, the younger participant became an outlier. Since individuals in different age groups were likely to have different media use habits, we made the decision to focus on middle-aged and older participants and excluded the younger participant from data analysis. In the end, 10 women and 10 men remained in the sample. The age of participants ranged from 41 to 72, with an average of 52. Participants were mostly well-educated (two with doctorates, six with master's degrees, three with bachelor's degrees, six with associate degrees or attended college/technical schools, and two graduated high school) and generally belonged to the middle class. In terms of geographic location, 15 were from a southern state, and the other five were from three different states in the United States. We gave each participant an alias to protect their identity (see Table 1 for aliases and demographic information of participants).

**Table 1:** Demographic Information of Participants.

	**Alias**	**Sex**	**Age**	**Education**
1	Ms. Antonio	F	48	Master's
2	Ms. Jay	F	51	Master's
3	Ms. Callie	F	50	Master's
4	Mr. Lindy	M	70	Some college, associate
5	Ms. Breesky	F	48	Ph.D.
6	Ms. Burrus	F	50	Master's
7	Ms. Tuckerton	F	42	Bachelor's
8	Ms. Aurora	F	45	Associate
9	Mr. Bass	M	52	Technical school
10	Ms. Delta	F	65	Ph.D.
11	Mr. Christy	M	72	Bachelor's
12	Mr. Brothers	M	47	Some college
13	Mr. MeHarry	M	60	Master's
14	Mr. Davenport	M	50	Some college
15	Mr. Antonio	M	52	Bachelor's
16	Ms. Booth	F	60	Some college
17	Mr. Phillips	M	42	High school
18	Ms. Aldine	F	41	Master's
19	Mr. Tony	M	49	Associates
20	Mr. Fontana	M	60	High school

### Data Collection

Semi-structured interviews were conducted. To answer RQ1, we asked questions such as “Which news source do you prefer when you want to get information during the COVID-19 pandemic,” “why do you prefer it over others,” “Do you use social media (if yes, how frequent) to get information during this period,” “What kind of information do you find most useful.” To answer RQ2, we asked questions such as “How will you judge if the information you get is credible.” Additional questions were asked, but answers to these questions were not reported in this article. These questions were adapted based on Tang and Zou (18) and Zou and Tang (28).

The second author conducted all the interviews. She was an African American doctoral student and has personal connection to the community studied in this study. She also had extensive experience with conducting qualitative interviews. The connection between the interviewer and interviewees made the latter more at ease and more willing to tell their real thought and feelings. The interviews were conducted over the phone or Facetime since data collection was completed in late March and early April 2020 when the participants were under social distancing order. Interviews typically lasted between 15 and 40 min. We recorded all interviews with participants' permission and used Otter.ai Voice Notes to transcribe the recordings automatically. The second author listened to all the interviews and manually corrected the transcripts. In the end, 254 single-spaced pages of transcribed interviews were used in data analysis.

### Data Analysis

We used the phronetic iterative approach for data analysis. The phronetic iterative approach is a novel method of qualitative data analysis that allows researchers to identify themes and subthemes informed by existing theories and to discover new themes and subthemes in a manner consistent with the grounded theory building approach (29). First, open coding was conducted to identify recurring concepts based on theories of information seeking and scanning and the grounded theory building approach. Second, axial coding was undertaken to establish the relationship among different concepts (e.g., CNN was often trusted because it was considered factual). Negative case analysis was conducted on cases that did not fit into the general patterns of relationships. Negative case analysis is a method to ensure the validity of the interpretation of qualitative data by analyzing outlier data (30). For instance, while most of the participants stated that they primarily relied on cable news channels such as the CNN and MSNBC, a couple of participants mentioned they watched Fox News. We paid especial attention to these negative cases to see if the rationale of choosing Fox News was the same as the motivation in choosing more liberal cable news channels. Conducting negative analysis allowed theoretical generalization based on the full range of data collected (30). Steps 1 and 2 described above were repeated in an iterative manner until we reached the themes and sub-themes based on our research questions. The first and third authors conducted the initial data analysis, and the second author confirmed the interpretation.

## Results

### RQ1: Seeking and Scanning Information About COVID-19

Overall, our participants primarily relied on information scanning for COVID-19 related information, utilizing TV and social media. Some also engaged in information seeking by reading news websites, using search engines, etc.

#### Information Scanning

Information scanning was the primary mode through which our participants obtained information about COVID-19. Information scanning happened when individuals were exposed to COVID-19 related information during their regular media consumption without consciously looking for it. It included watching TV, using social media, and listening to one's family and friends.

##### TV

Our participants primarily relied on TV for COVID-19 related information. They often turned to cable news such as CNN, Fox News, and MSNBC for national coverage and local news channels operated by ABC, CBS, and NBC for information relevant to their cities and neighborhoods. Among cable news, CNN was by far the most used. Thirteen out of twenty participants mentioned they watched CNN for COVID-19 related information. Participants chose one cable news channel over others for several reasons. Some chose CNN because they believed that CNN was more neutral or more factual. For instance, Ms. Breeskey (P5, 48) said, “they're kind of neutral [.] in my opinion. I know if you flip it to like other news stations, it may not. It may be a total opposite of what they're saying.” Some participants chose a cable news channel because it was more aligned with their political beliefs. For instance, Mr. Christy (P11, 72) explained why he chose CNN over Fox News, saying, “I prefer CNN because they typically cater to the democratic public, rather than Fox news because they typically rely on the Republicans.” Similarly, Ms. Aldine (P18, 41) preferred MSNBC because “I feel like my views line up with most [of the personalities] on MSNBC.” Unlike CNN and MSNBC, Fox News was used to provide an alternative perspective.

In terms of local news stations, local TV channels operated by ABC, NBC, and CBS were all mentioned by our participants. In contrast to their rationale in choosing cable news, many of our participants reported that they watched local TV stations out of habit. For instance, Ms. Aldine (P20, 41) watched ABC13 for local news because “no real rhyme or reason. My grandmother was always watching Channel 13. Okay, it just kind of stuck with me, so I just prefer it. I mean, I like the reporters and everybody there.” When asked why she used Channel 13, Ms. Burrus (P6, 50) said, “I wouldn't say a relationship with the media, but I guess I've developed [a relationship] with people (hosts) on the news media that I like, and I can trust.” Occasionally, participants stated that they chose a particular local TV channel because of the quality of coverage.

##### Social Media

While almost all participants heavily used television for information about the COVID-19 pandemic, their social media use was more varied. Some used social media extensively, checking them many times a day. For example, Mr. MeHarry (P13, 60) said he had been checking social media at least two or three times a day but had “slaked up” a little recently. He now checked social media once a day because the “information does repeat itself quite frequently now.”

Seven participants explicitly stated that they did not use social media for COVID-19 related information, or they rarely used social media for such information. They offered several explanations for this decision. Some did not deem the news on social media to be credible. Ms. Antonio (P1, 48) explained, “you get a lot of opinions, and people copying and pasting from their other resources, and a lot of times it's just a lot of miscommunication, or the information may not be accurate.” Others stated that they only used social media to stay connected to their family and friends. For instance, Ms. Booth (P16, 60) said, “I don't utilize social media for news. I use social media to, maybe, connect with a family member that's living in a different state just to see how they're doing.”

Among those who used social media for COVID-19 related information, many mentioned that they primarily relied on push notifications. For example, Ms. Jay (P2, 51) said, “I use social media pretty much daily. And sometimes I may see things, news that [.] pop on one of the social media outlets that I'm looking at. I don't just seek to see what social media is saying about it, but while I'm on there, I generally see things going on via the newsfeed.” Ms. Callie (P3, 50) made almost precisely the same comment. “I wouldn't say I'm going to social media just to look for that [information], but it pops up.”

##### News Websites and Apps

Several participants used news websites and news apps through their mobile phones. Some preferred to use news websites such as Yahoo News. Ms. Bass (P9, 52) explained, “Because sometimes I feel that Yahoo gives me more in-depth things that other sites don't. Sometimes I feel like they might go more in-depth than NPR.” Some participants accessed their preferred TV channels through mobile phone apps. For instance, Ms. Booth (P16, 60) said, “I have my local news app on my phone. So, it'll come across the phone like a headline or breaking news. You know, or they'll say okay a live update, I can just push that and it'll bring me to what's taking place.”

##### Family Members and Other Interpersonal Contacts

Some participants reported that they relied on their family members and other interpersonal contacts for credible information about the COVID-19 pandemic. For instance, Ms. Antonio (P1, 48) said, “I really use my husband because, um, he, you know works for this really big company, and he is staying on top of the news, and what's going on in the world and an economy is a big deal and a big part of his job. So he's always giving me great sources […]. And so, I feel comfortable receiving it from him because of the industry that he's in.”

#### Information Seeking

Information seeking happened when individuals went out of their way to look for a piece of information. Some participants sought information by checking governmental websites. Some used search engines to look for specific information. However, in general, information seeking was limited among our participants.

##### Governmental Websites

Governmental websites were used by a few participants to access credible information about the COVID-19 pandemic. When asked which news source he preferred when looking for information during the pandemic, the first response given by Mr. Fontana (P20, 60) was “dot gov. The government.” He explained, “cause it speaks of accuracy. It speaks about what they have done, and you know they are behind, but it ain't falsification.” Similarly, Ms. Aurora (P8, 45) preferred the CDC website because “it is assumed that they are experts getting out the pertinent information.”

##### Search Engine

Four participants reported that they used search engines such as Google and Yahoo when they wanted to find information about a specific topic. Having these search engines available increased our participants' confidence that they were capable of finding high-quality information about the pandemic. Mr. Tony (P19, 49) said, “I'm not moderately skilled. I'm very skilled. I know what I'm looking for. I can Google if I need to go look at the news, the local news channel and see what they're saying about COVID 19 today.” However, even though search engines are readily available, some participants just did not feel the need to search for information. For instance, Mr. Lindy (P4, 70) said, “I am very confident in finding information, but I just didn't look any further.”

### RQ2: Information Evaluation

RQ2 asked how African American individuals evaluated the information they received about COVID-19. A few participants mentioned they did not try to assess the quality of the information they received. When asked what she did to evaluate the information she received, Ms. Tuckerton (P7, 42) said, “I don't know for a fact. I just go with it and hope that it is accurate.” However, most participants did use at least one of the several common strategies of information evaluation, including evaluating source credibility, comparing news from multiple sources, fact-checking through search engines, and appealing to a higher existence (praying).

#### Evaluating the Credibility of Information Source

Source credibility was the most often used cue for individuals to judge the quality of the information they received, and it required the least effort. Almost all participants discussed sources that they perceived to be credible, including cable news, local TV stations, trusted TV and radio hosts, and the government. Some participants also expressed that they relied on their trusted family members or contacts for information.

#### Comparing News From Multiple Sources

Many participants reported comparing the news from multiple sources, and if they saw the same information from several sources, they considered it to be credible. Mr. Brothers (P12, 47) discussed how he processed the 5G conspiracy theory, saying, “Well, when I do the search, I look at three to four articles that they're saying the same thing. I checked that off as being credible. But if I hear something and comprehend it. Not a lot of people are talking about it. An example is the 5G. So, I hear a lot of people, some people talking about the 5G had something to do with it, but not a lot of people talking about it or dismissing it. So, I just let that one slide.”

Sometimes, participants paid particular attention to news sources with different political leanings. For instance, Ms. Antonio (P1, 48) said, “You know what I do? I cross-reference. So, for instance, CNN tends to be a little more liberal. […] A lot of times, they're being a little biased about how the government is responding because maybe they're not a fan of Trump. So, what I do, I'll go back and forth. I'll watch Fox. I'll watch CNN. I try not to stay with just one particular side.”

#### Fact-Checking Through Search Engines

Several participants reported fact-checking the information they received about COVID-19, especially information from social media. Mr. Christy (P11, 72) said, “But I typically do the research if it's an interesting topic and I want to know more about the topic […], then I will dig a bit further, and not necessarily on that platform, but through internet searches or through other outlets. To find out more about that particular topic, so I don't know that the information they provide is credible. But I do dig a bit more to find out if the information is legitimate.”

#### Praying

Religion was an important part of our participants' coping strategies facing the COVID-19, and it was occasionally brought up as a way to ascertain the quality of the information participants received. Mr. Bass (P9, 52) stated that when he was not sure whether a piece of news was accurate or not, he would turn to his faith, saying, “That's not always easy to know if it's accurate or not, you know. You have to be honest with yourself because there are rumors out there […]. So, you have to learn how to discern what's going on. What is that, that's why some of us are actually getting on our knees and praying every day and asking God for discernment of what we're actually looking at and listening to make sure that whatever information we're getting is accurate and precise.”

## Discussion

Our study shows that middle-aged and older African American individuals often use credible sources for COVID-19 related information during the early months of the pandemic, even though they acquire such information primarily through information scanning instead of information seeking. Like other groups, they typically evaluate the information they have through heuristic processing.

### Information Scanning Based on Media Consumption Routines

Interviews with middle-aged to older African American individuals showed that most of them acquired information about the COVID-19 pandemic through information scanning via their routine media consumption channels. Cable news, especially CNN and MSNBC, were frequently used either because they were more in line with our participants' political beliefs or because they were considered to be more factual and unbiased. Our participants overwhelmingly relied on local television news stations operated by ABC, NBC, or CBS for COVID-19 updates and information in the local area. They typically chose local news stations out of their media consumption habit and usually did not go out of their way to use other sources. Surveys conducted in the UK showed that broadcast media consumption was positively correlated with health-protective behaviors (31). Our participants also reported using and trusting governmental sources such as the CDC and governmental websites. This is consistent with Oh et al. (27)'s analysis of HINTS data. A survey study on Black American's trust in COVID information sources also shows that they are significantly more likely to trust the government and mainstream media than non-black Americans (32). We have reasons to believe that our participants have access to relatively high-quality information about the pandemic offered by traditional media channels, which is conducive to proper protection and prevention behaviors.

At the same time, Allington et al. (31) also found that social media use was positively correlated with belief in conspiracy theories. Furthermore, a survey conducted in China showed that spending more than 2 h daily on COVID-19 news via social media was associated with probable anxiety and depression (33). While some of our participants do use social media for health information related to the COVID-19 pandemic, many participants were cautious about the quality of the information they received through social media. This is in line with the finding of a similar study conducted in China, which finds that while young people almost exclusively rely on social media for COVID-19 related information, middle-aged and older adults primarily use traditional media, especially TV (18). Some participants intentionally chose to reduce their exposure to social media in order to keep a positive mindset. Consistent with the RISP and PRIP models, our participants reported a high level of perceived severity and susceptibility of the risk pushed them away from frequent information-seeking by triggering their negative emotions.

In general, middle-aged to older African American individuals in our sample seldom engaged in active information-seeking during the COVID-19 pandemic. Overall, they do not feel the need to seek information actively. By contrast, some participants preferred to consider praying as a primary coping strategy, thus decreasing their motivation in taking the initiative to seek information.

### Evaluation of Information About COVID-19

In general, our participants appear to have limited motivation and capability in differentiating rumors. They primarily use heuristic processing to evaluate the credibility of the COVID-19 related information they receive by checking the sources' credibility and deciding if the information is carried in multiple channels. This echoes the findings of a similar study in China (28). Such strategies allow them to make quick decisions about the validity of a piece of information. These heuristic processing strategies will usually suffice for those African American individuals who primarily rely on cable news and local TV news for COVID-19 related information. However, they might be inadequate in helping people identify the misinformation and rumors about the COVID-19 that flood social media platforms. Only a few reportedly use fact-checking through search engines to validate the specific claims made in news articles or messages. Fact-checking could be an effective strategy that helps all participants evaluate COVID-19 related information from less authoritative sources.

### Unique Patterns of Social Media Uses

Unlike previous studies that suggest increased social media use for information seeking and exchanging among other demographic groups [e.g., (18)], our findings show that middle-aged and older African Americans are less likely to use social media for COVID-19 related information. Although most of them have social media accounts, they mainly consider social media as an important channel to communicate with family members instead of sources of pandemic-related information. Furthermore, most participants said they seldom posted or reposted any messages about COVID-19 during the pandemic. Instead, they sent private messages to their family and friends. According to the Uses and Gratification Theory, individuals' media choices are usually linked with their particular needs (34). Our study suggests that participants' social integrative needs become the main goal for social media use, compared to other needs (e.g., cognitive needs, tension-free needs, etc.).

### Practical Implications

Our study finds that middle-aged and older African American individuals overwhelmingly rely on cable news (CNN) and local television stations (ABC, CBS, and NBC) for COVID-19 related information, and they have high trust in these sources. This means that traditional TV channels are probably the most effective way to reach this particular demographic group in terms of risk communication about the COVID-19 pandemic and other public health crises in the future. For the demographic group studied in the current study, knowledge deficiency is probably not a contributing factor to the disparity related to COVID-19. In particular, since religion plays an essential role in African American's coping with this health risk, health organizations could integrate faith-based content into health messages to attract this group's attention.

## Limitations and Directions for Future Research

Our participants were mostly middle-aged or older African American individuals with relatively higher education. Younger or less educated members of the African American community might have different patterns of information acquisition and evaluation. For instance, younger individuals may rely more on social media for information. Additional research isF needed to understand these demographic groups and how they acquire and evaluate information about COVID-19. Secondly, our data collection occurred between late March and early April of 2020. It was a period when the country was on high alert while the number of infections was relatively low. Since individuals' information needs and information usage change significantly during different stages of a public health crisis (35), follow-up studies are needed to provide a comprehensive understanding of African-American individuals' media use for COVID-19 related information. Finally, a future survey study should examine the relationship between media use patterns, knowledge, and protective behaviors.

## Data Availability Statement

The original contributions presented in the study are included in the article/supplementary material, further inquiries can be directed to the corresponding author/s.

## Ethics Statement

The studies involving human participants were reviewed and approved by Institutional Review Board, Texas A&M University. Written informed consent for participation was not required for this study in accordance with the national legislation and the institutional requirements.

## Author Contributions

LT and WZ conceptualize the study. FY collected data. LT wrote the manuscript. All authors contributed to data analysis.

## Conflict of Interest

The authors declare that the research was conducted in the absence of any commercial or financial relationships that could be construed as a potential conflict of interest.

## Publisher's Note

All claims expressed in this article are solely those of the authors and do not necessarily represent those of their affiliated organizations, or those of the publisher, the editors and the reviewers. Any product that may be evaluated in this article, or claim that may be made by its manufacturer, is not guaranteed or endorsed by the publisher.

## Footnotes

^1^We used the term African Americans in this paper unless the term black/Black was used in the literature cited or used by interview participants.
